# Trends in Stranding and By-Catch Rates of Gray and Harbor Seals along the Northeastern Coast of the United States: Evidence of Divergence in the Abundance of Two Sympatric Phocid Species?

**DOI:** 10.1371/journal.pone.0131660

**Published:** 2015-07-22

**Authors:** David W. Johnston, Jaime Frungillo, Ainsley Smith, Katie Moore, Brian Sharp, Janelle Schuh, Andrew J. Read

**Affiliations:** 1 Division of Marine Science and Conservation, Nicholas School of the Environment, Duke University of Marine Laboratory, 135 Duke Marine Lab Rd., Beaufort, NC, 28516, United States of America; 2 Integrated Statistics, 16 Sumner Street, Woods Hole, MA, 02543, United States of America; 3 International Fund for Animal Welfare, Marine Mammal Rescue and Research Program, 290 Summer St., Yarmouth Port, MA, 02675, United States of America; 4 Animal Rescue Program, Sea Research Foundation, 55 Coogan Blvd., Mystic, CT, 06355-1997, United States of America; Aristotle University of Thessaloniki, GREECE

## Abstract

Harbor seals and gray seals are sympatric phocid pinnipeds found in coastal waters of the temperate and sub-Arctic North Atlantic. In the Northwest Atlantic, both species were depleted through a combination of subsistence hunts and government supported bounties, and are now re-occupying substantial portions of their original ranges. While both species appear to have recovered during the past 2 decades, our understanding of their population dynamics in US waters is incomplete. Here we describe trends in stranding and bycatch rates of harbor and gray seals in the North East United States (NEUS) over the past 16 years through an exploratory curve-fitting exercise and structural break-point analysis. Variability in gray seal strandings in Southern New England and bycatch in the Northeast Sink Gillnet Fishery were best described by fitting positive exponential and linear models, and exhibited rates of increase as high as 22%. In contrast, neither linear nor exponential models fit the oscillation of harbor seal strandings and bycatch over the study period. However, a breakpoint Chow test revealed that harbor seal strandings in the Cape Cod, Massachusetts region and harbor seal bycatch in the Northeast Sink Gillnet Fishery increased in the 1990s and then started declining in the early to mid-2000s. Our analysis indicates that ongoing variation in natural and anthropogenic mortality rates of harbor and gray seals in the NEUS is not synchronous, and likely represents diverging trends in abundance of these species as they assume new roles in the marine ecosystems of the region.

## Introduction

Harbor seals (*Phoca vitulina*) and gray seals (*Halichoerus grypus*) are sympatric throughout their ranges in temperate and sub-arctic coastal waters of the North Atlantic. Both species previously formed large colonies along the east coast of North America from Labrador to Cape Hatteras [1–3]. During the 19^th^ & 20^th^ centuries populations of seals in this region were depleted by subsistence hunting [4] and government-sponsored bounty programs [5]. In 1972, the Marine Mammal Protection Act provided both species almost complete protection in the northeastern U.S. (NEUS) and both species are currently managed as single stocks with some level of cross-boundary exchange with Canada. In the four decades following enactment of this statute, both gray and harbor seals have grown in abundance and reoccupied substantial portions of their original ranges [4]. This recovery has occurred in a greatly changed seascape in which fisheries resources have been depleted by over-harvest and shorelines have become urbanized.

The gray seal population is continuing to recover throughout the NEUS, increasing in abundance and establishing new breeding colonies. While no true abundance data for gray seals in the NEUS exist, limited beach counts and pup counts indicate that the population is growing rapidly [4,6]. However, the situation for harbor seals is less clear. From 1997 to 2001 the harbor seal population grew by more than 27% and reached almost 100,000 individuals [7]. However, the most recent survey, conducted in 2011, yielded an estimate of only 70,000 seals [7]. The coefficients of variation for these point estimates are large, and surveys have not been comprehensive, so it remains unclear if the population has indeed declined [7]. Unlike the situation for gray seals, however, there is no evidence to suggest that the population of harbor seals is continuing to increase in the NEUS. Our understanding of the dynamics of both populations is incomplete because they have been the subject of relatively little attention from the scientific and management communities.

Other harbor seal and gray seal populations in the North Atlantic have exhibited divergent trends in abundance in recent years. For example, harbor seals on Sable Island, off the coast of Nova Scotia, have declined precipitously in recent decades [8,9] and are now largely absent from this colony. In contrast, the number of gray seals using Sable Island grew at rates of up to 13% annually [10] and only recently has this rate of growth slowed. Similar patterns are evident in the United Kingdom where harbor seals are declining and sympatric gray seals are continuing to increase in abundance [11,12].

Monitoring population trends for seals and other marine vertebrates is challenging [13], particularly when it is difficult to generate a time series of precise estimates of abundance. In such cases we must rely on indices that reflect the true trajectories of populations, such as beach counts or other observational data [14], to determine demographic status. Such indices may not yield robust estimates of abundance, but can be used to establish the general status of marine animal populations and their relative trajectories [15]. For example, the marine mammal stranding record is known to reflect the diversity and relative abundance of cetaceans in coastal waters [16] and can provide a relatively low-cost index of population status [17]. Recent studies have linked changes in stranding rates of pinnipeds to both long and short-term environmental perturbations [18,19] and to variation in local abundance [20].

In the present study we employ observational data to investigate potential differences in the population biology of seals in the NEUS. Specifically, we assess trends in stranding and bycatch rates of harbor and gray seals in the NEUS over the past 16 years to provide context on changes in their populations in the region. The consistent patterns revealed by these analyses provide insight into how populations of these two species are diverging, and perhaps interacting, as they assume new roles in the marine ecosystems of the NEUS.

## Methods

### Stranding data

**Fig 1:**
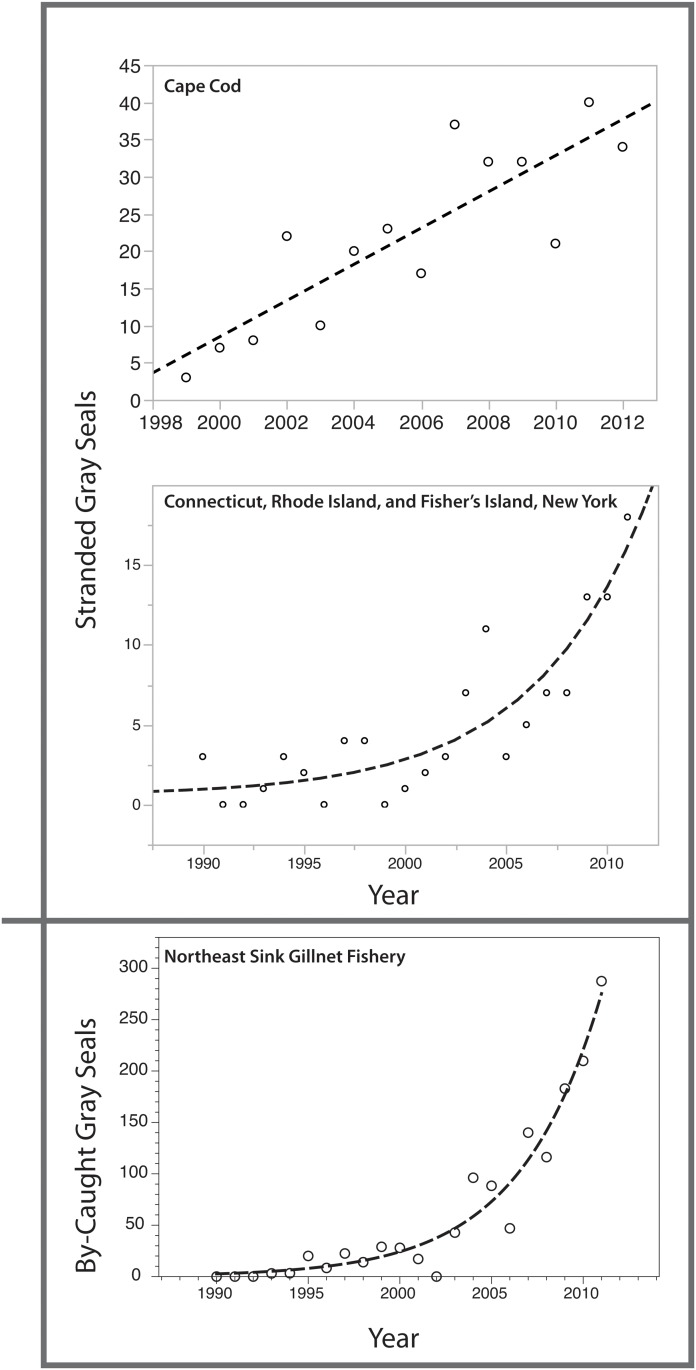
Strandings of gray seals in the Cape Cod and Connecticut, Rhode Island, and Fisher’s Island, New York regions, and bycatch for the Northeast Sink Gillnet Fishery. Yearly values indicated by open circles, linear and exponential model fits indicated by dashed lines.

We extracted stranding data from datasets maintained by International Fund for Animal Welfare (IFAW)’s Marine Mammal Rescue and Research Program and Mystic Aquarium’s Marine Mammal and Sea Turtle Stranding Program. These organizations are members of the National Marine Fisheries Service (NMFS) Greater Atlantic Region Stranding Network. The IFAW responds to strandings on more than 700 miles of coastline of Cape Cod and Southeastern Massachusetts and Mystic Aquarium responds to strandings on more than 1,000 miles of coastline of Connecticut, Rhode Island, and Fisher’s Island, New York. The IFAW data set included all harbor and gray seal strandings from 1999 to 2012 (Fig 1) and Mystic Aquarium included strandings from 1990 to 2011. These organizations respond to strandings along the most highly urbanized portions of the east coast of the U.S., and have had relatively consistent coverage during the timeline of the present study.

Every response to a live or dead stranded seal generates a basic set of information referred to as ‘Level A data’. These data capture the basic details of the stranding event, including the species and the condition of the specimen, date of the stranding, physical evidence of ‘human interaction’ (such as net marks or gunshot wounds) and location. Trained personnel did all assessments of condition and human interaction according to detailed protocols [21]. We used these Level A data to assess inter-annual trends of gray and harbor seal strandings in the NEUS. A small number of animals included in the Cape Cod data were determined to have died as a result of direct human interaction (HI), and these animals were not used in our analyses. There was evidence of HI in 4 cases in the Connecticut, Rhode Island, and Fisher’s Island, New York dataset, but these interactions were not determined as cause of death, so these data were included in our analysis.

### Bycatch data

We also obtained annual estimates of the number of gray and harbor seals killed in the Northeast Sink Gillnet Fishery between 1990 and 2011 from annual Stock Assessment Reports (SARs) generated for the two species and published by the National Oceanic and Atmospheric Administration [6]. The SARs include estimates of the total number of animals killed each year in the Gulf of Maine; these estimates are derived as the product of an observed rate of bycatch and a measure of total fishing effort to address potential bias introduced by variation in fishing effort. By-catch rate is estimated from direct observations made by independent on-board observers of the number of seals killed taken in a sample of the fishery [6]. Total by-catch is then extrapolated by multiplying this rate with a measure of total yearly fishing effort, expressed as tons of fish landed each year, and presented in Appendix III of the annual SARs volumes [6,7]. Landings data were missing for the early portion of the time series, so to correct these values we used the mean of the entire data set as a proxy.

### Trend Analysis

To explore changes in stranding and bycatch rates over time, we conducted an exploratory curve-fitting regression analysis on both harbor and gray seal time series using JMP Pro Version 10.0. We fit a series of linear and exponential regression models to stranding and bycatch data using JMP Pro Version 10.0 and DataGraph version 3.2.

### Structural Breakpoint Analysis

We used a Chow test [22] in the R statistical programming environment [23] to look for significant discontinuities in the stranding and bycatch data time series that were not well described by linear or exponential models. A significant Chow test indicates that a structural change exists in the data trajectories at a specified break point [22]. We set the break point for each data set to the year in which harbor seal strandings reached a maximum (2004 for IFAW and 1996 for Mystic and 2000 for bycatch data). The Chow test requires output from three linear models run on the time series of data: a restricted linear model on the entire dataset, an unrestricted linear model on all data after the break point, and an unrestricted linear model on all data prior to the break point [22]. The Chow test is calculated using the following formula:

$$\frac{\left(SSR_{r}-SSR_{u}\right)/k}{SSR_{u}/\left(n-2k\right)}\;\sim \;F_{k,n-2k}$$

Where *SSR*_*r*_ is the sum of squared residuals of the restricted model, *SSR*_*u*_ is the sum of squared residuals from the unrestricted models, *k* is the number of coefficients, and *n* is the total number of observations.

## Results

**Fig 2:**
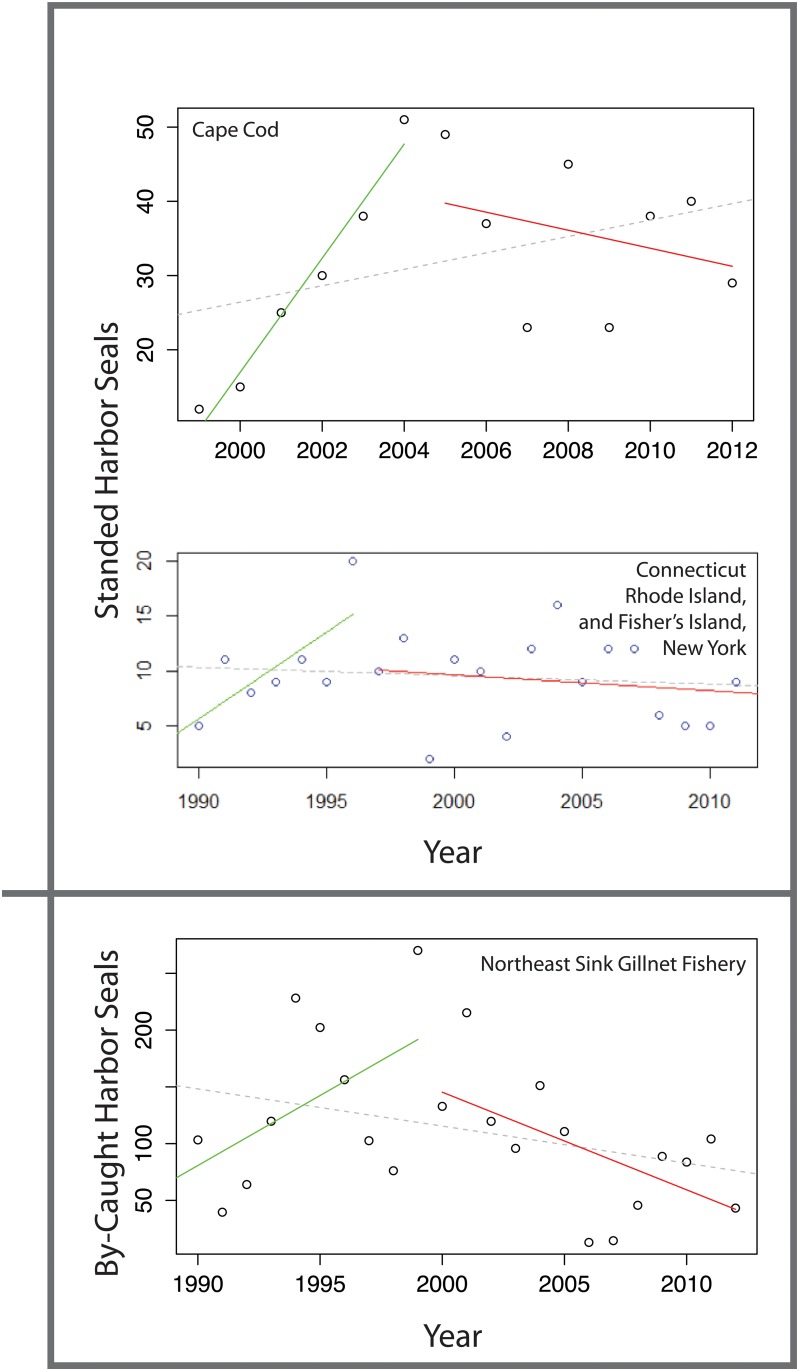
Strandings of harbor seals in the Cape Cod and Connecticut, Rhode Island, and Fisher’s Island, New York regions, and bycatch for the Northeast Sink Gillnet Fishery. Yearly values indicated by open circles, pre-breakpoint linear model fits indicated by green lines, post breakpoint linear model fits indicated by red lines. Overall regression indicated by dashed gray lines.

Trends in stranding and bycatch rates for gray and harbor seals in the NEUS are clearly divergent. Gray seal strandings and bycatch are increasing (Fig 1), whereas harbor seal strandings and bycatch are either declining or, at best, stable (Fig 2). The details of regressions fit to stranding and bycatch data are presented in Table 1. For two of the three gray seal datasets, 3 parameter exponential models provided the best fit to the data, indicating that gray seal bycatch and standings in the Southern New England region have increased at rates between 18 and 22 percent since the early 1990s. Changes in the stranding rates of gray seals in the Cape Cod region were best modeled with a positive linear regression, largely because the Cape Cod dataset is shorter in duration than the other two datasets (Fig 1).

**Table 1:** Details on linear and exponential models fit to strandings of harbor and gray seals in the Cape Cod and Connecticut, Rhode Island, and Fisher’s Island, New York regions, and bycatch for the Northeast Sink Gillnet Fishery.

Species	Location	Period	Model	Slope/Rate	R-square	Chow Test P value
**Harbor Seals**	Cape Cod	Early	Linear	7.86	0.96	
		Late	Linear	-1.2	0.10	0.01
		Overall	Linear	1.1	0.15	
	CT, RI, NY	Early	Linear	1.57	0.52	
		Late	Linear	-0.14	0.15	0.25
		Overall	Linear	-0.02	0.02	
	NE Sink Gillnet	Early	Linear	12.3	0.24	
		Late	Linear	-8.6	0.35	0.04
		Overall	Linear	-3.3	0.11	
**Gray Seals**	Cape Cod	Overall	Linear	0.59	0.61	n/a
	CT, RI, NY	Overall	Exponential	0.18	0.80	n/a
	NE Sink Gillnet	Overall	Exponential	0.22	0.94	n/a

The Chow test revealed a significant break (P < 0.01) in both the bycatch and Cape Cod stranding datasets for harbor seals (Fig 2, Table 1). In both datasets the number of harbor seals climbed until the early 2000s, after which stranding and bycatch rates began to decline. The Mystic stranding data does not provide as clear a picture, with only a slight decline after an initial peak of strandings much earlier in the data set (1996). However, if the highest stranding rate in the time series—1996—is excluded from the dataset as an outlier, the revised dataset peaks in 2004. Regardless, the breakpoint analysis does not indicate a significant change in the stranding rates across the time series for harbor seal strandings in Connecticut, Rhode Island, and Fisher’s Island, New York (Fig 2, Table 1).

## Discussion

Taken together, the three datasets provide a consistent picture of diverging seal mortality that likely reflects broad patterns in the abundance and distribution of these two phocid species. The dramatic rise in gray seal bycatch and strandings is not surprising, considering increasing trends in beach and pup counts in the region [4,6], and is consistent with observed recent increases in gray seal populations in other areas such as Sable Island. It is important to note that the there is a strong demographic connection between gray seals on Sable Island (and elsewhere in eastern Canada) and those in the NEUS, driven by movements of animals from Canada to the U.S. [24]. Similarly, immigration of gray seals from established colonies in the North Sea has contributed strongly to the rapid recovery of gray seals in the waters of the Netherlands [25].

The initial rise in bycatch and strandings of harbor seals in all three of our datasets likely reflects the initial recovery of this species in the NEUS. The subsequent decline in bycatch and stranding rates of harbor seals starting in the early 2000s is disconcerting. As noted above, the estimated abundance of harbor seals in the NEUS dropped by nearly 30,000 between 2001 and 2011 [7]. The confidence intervals for these estimates are large, and it remains unclear if these latest survey results reflect a true decline in the abundance of harbor seals [7]. This is a common issue associated with monitoring marine mammal populations in general [13]. Nonetheless, the concomitant decline in stranding and bycatch rates of harbor seals presented here provide support for an apparent decline in abundance, as suggested by these survey results [7]. If the trends in bycatch and stranding data described in the present analysis reflect broad patterns in the living population, then it is likely that harbor seals in Southern New England are in decline. The timing of changes in strandings and bycatch rates in harbor seals is consistent with the timing of estimates generated from surveys, supporting this inference. Both stranding and by-catch rates for harbor seals in Southern New England begin to decline sometime in the early to mid-2000s, between the two most recent surveys.

Fine-scale changes in harbor seal habitat use may provide some context for the patterns described here. For example, species composition at several seal haulouts in Cape Cod has changed dramatically over the same time frame. Some haulouts in Cape Cod that were formerly populated with harbor seals now only support growing numbers of gray seals [26,27] and a long-term study in northern Maine indicates that harbor seals were spending less time at traditional haulouts in Casco Bay as early as 2003 [28]. In general, however, there has been very little systematic research on the ecology and behavior of these two species at scales in the NEUS that could help to explain this possible divergence in both their bycatch and stranding rates.

Our results are consistent with changes in the status of harbor seal populations in many other locations where they are sympatric with gray seals. Harbor seals have declined dramatically elsewhere in the Northwest Atlantic over the past few decades, and this decline has been best studied on Sable Island. Harbor seals were abundant on Sable in the 1980s, representing the largest colony in Eastern Canada [8]. Since that time the number of harbor seals using Sable Island has dropped dramatically and the species is now essentially absent from the island. Harbor seal pup production declined dramatically during this period. For example, in 1989, 600 pups were born on Sable Island, whereas in 1997 only 30 pups were born [8]. The abundance of harbor seals throughout much of Scotland has also declined recently, and declines have been noted in southern portions of the UK as well, associated to some extent with the lasting effects of phocine distemper (PDV) outbreaks, amongst other causes [11,29,30]. This phenomenon is not universal, as harbor seals have recovered from die-offs in the Wadden Sea and continue to do so (data available at: http://www.waddensea-secretariat.org/monitoring-tmap/topics/marine-mammals.).

### What is the cause of diverging rates?

Is it possible that harbor, but not gray seals, have reached carrying capacity in the NEUS? Gray seal abundance grew dramatically on Sable Island during 1960 to 1997 [10] and now appears to be reaching the limits of growth at that location. In Southern New England, beach counts of gray seals are still increasing (fuelled, in part, by emigration of animals from Sable Island), and this growth is reflected in the increase in by-catch and strandings rates of gray seals presented here. Considering the effects of density dependence on seal populations [31], we would expect to see rapid growth in the harbor seal population followed by growth and stabilization in stranding rates of juvenile animals and a stabilization of bycatch rates over time if the population was approaching carrying capacity. This may be true for southern New England, as reflected by the Mystic stranding data set. However, the patterns in harbor seal strandings from Cape Cod and bycatch rates from across the Gulf of Maine presented here deviate from these expectations, as both declined in the early to mid-2000s. These findings, coupled with the recent lower point estimate for harbor seals, do not suggest that harbor seals are approaching their carrying capacity in the NEUS.

Is competition amongst seal species a driving factor in these divergent datasets? Several other studies have hypothesized that gray seals may outcompete harbor seals where they are sympatric [9]. Gray seals are becoming more common at traditional harbor seal haulouts, but it does not seem that direct terrestrial competition for space is currently occurring, although gray seals can affect interactions amongst harbor seals at mixed haulouts [32]. Furthermore, the breeding and molting periods for these two species are offset in time, reducing competition for space during these critical periods. The potential for competition for prey resources is difficult to assess, as very little work has focused on the foraging ecology of gray and harbor seals in the NEUS. The diet of harbor seals and gray seals does overlap and may lead to competition for prey in some locations [9,10], although temporal and spatial overlap in the movements and at-sea foraging behavior of these two species remains largely unstudied in the NEUS.

Is there greater predation pressure on harbor seals in the NEUS? The abundance of white sharks in the North Atlantic is increasing [33], and there are indications that growing seal populations in the NEUS are attracting greater numbers of these predators into coastal waters [34]. A combination of shark-related mortality and competition for prey has been implicated in the decline of harbor seals on Sable Island [9,35]. It remains unclear if sharks in the US Northeast preferentially prey on harbor seals. The stranding data for Southern New England during 2000 to 2006 [36] illustrates that the total number of harbor seals stranded as a result of shark attacks was twice that of gray seals, although the proportions of animals stranded with evidence of shark predation in both harbor and gray seals were similar (10% and 7% respectively). There are indications that gray seals can also prey directly on harbor seals pups [37], although this has not been reported for the NEUS, and no links to population levels effects have been established where it has been observed.

Are changing environmental conditions contributing to divergence in stranding and bycatch rates for these two species? The effects of environmental changes on marine mammal populations can contribute to shifting patterns in strandings and bycatch. For example, both short and long-term climate effects on sea ice in the breeding regions of harp seals correlate with stranding rates of this species in the NEUS [18,19]. There are currently no obvious environmental signals that may directly explain the exponential increases in gray seal strandings and bycatch observed in the NEUS. However, it is possible that the patterns revealed in regional harbor seal bycatch in the present study represent a recent shift in the distribution of these animals, perhaps in response to ongoing warming [38]. This is not, however consistent with observed changes in harbor seal bycatch in the NEUS, which integrate information across a much wider latitudinal range. Additionally, there are no indications that harbor seal abundance is climbing either north or south of the NEUS. Further research is required to assess links between changing climate factors and the natural and anthropogenic mortality rates of these species in the NEUS.

Do harbor seals and gray seals differ in their vulnerability to infectious disease? If harbor seals were more vulnerable, we would expect to see similar patterns of growth in stranding rates and bycatch rates in both species, followed by periodic disease-related peaks in strandings of harbor seals, as seen in other locations. There have been unusual mortality events (UMEs) for NEUS harbor seals in 2003, 2005, 2006 and 2011 [7] and the most comprehensive analysis of stranding data from 2000 to 2006 found that disease was the most prevalent factor associated with harbor seal strandings [36]. Historically, some strains of influenza A virus (IAV) have been linked to mass mortalities of harbor seals in the NEUS [39]. For example, during 1979–80 large numbers of harbor seals in the Cape Cod region died of pneumonia associated with H7N7 IAV infection [40], and other IAV strains have been linked to seal mortalities in the NEUS [39] and recent die-offs in Europe [41]. If the demography of the harbor seal population is being driven by disease, we would also expect a decline in bycatch as their abundance declines in the wild. Phocine distemper virus (PDV) outbreaks have been a major driver of observed declines in the abundance of harbor seals in the Southern UK and in the Netherlands, Denmark, Germany and Sweden, and seals appear vulnerable to this disease in some locations [42]. Interestingly, there were no UMEs for gray seals in the NEUS during this period and in other locations this species appears more resilient than harbor seals to the effects of PDV infections [43]. During 2000 to 2006, disease and human interactions were equally associated with gray seal strandings in the NEUS [36] and the bycatch and stranding rates presented here do not support the idea that gray seals are suffering from repeated PDV epizootics, or other infectious diseases stemming from influenza infections. Considering the available evidence, it seems that harbor seals may be more vulnerable to emerging infectious disease in the NEUS than gray seals. Experimental exposure to of both gray and harbor seals to H7N7 IAV produced different results [40]. Harbor seals presented disease symptoms similar to naturally infected seals, whereas gray seals presented no sign of infection. It should be noted that H7N7 IAV exposure to technicians by an infected seal resulted in cases of conjunctivitis linked to seal H7N7 [40]. In the NEUS, gray seals may be more socially gregarious than harbor seals, forming high-density aggregations of hundreds of animals. As such, they may have robust immune systems adapted to conditions at haulouts where rapid transmission of disease occurs. If this is true, gray seals may also represent a reservoir for pathogens that have stronger effects on sympatric harbor seals. Further research is required to assess variability in immune systems across phocid seals, how infectious disease is transmitted through their colonies, and what such factors may mean for the demography of these species [39].

## Conclusions

Stranding and bycatch rates for gray and harbor seals are diverging in the NEUS, with gray seal rates continuing to increase exponentially while harbor seals rates are declining or stable. We believe that these divergent patterns reflect changes in the demography of these two species as they re-occupy portions of their ranges. The declining stranding and bycatch rates for harbor seals is of particular concern considering the most recent survey results and the recent loss of harbor seals on Sable Island. Further research is required to determine the true trajectory of the harbor seal population in the NEUS and to determine what ecological factors are limiting harbor seal recovery.
